# Influence of Accelerated Weathering on the Mechanical, Fracture Morphology, Thermal Stability, Contact Angle, and Water Absorption Properties of Natural Fiber Fabric-Based Epoxy Hybrid Composites

**DOI:** 10.3390/polym12102254

**Published:** 2020-09-30

**Authors:** Krittirash Yorseng, Sanjay Mavinkere Rangappa, Jyotishkumar Parameswaranpillai, Suchart Siengchin

**Affiliations:** Department of Mechanical and Process Engineering, The Sirindhorn International Thai-German Graduate School of Engineering (TGGS), King Mongkut’s University of Technology North Bangkok, Bangkok 10800, Thailand; y.krittirash@gmail.com (K.Y.); mcemrs@gmail.com (S.M.R.); jyotishkumarp@gmail.com (J.P.)

**Keywords:** kenaf, sisal, epoxy, accelerated weathering, mechanical properties, water absorption

## Abstract

Epoxy-based biocomposites are a good alternative for metals in lightweight applications. This research has been focused on the effect of accelerated weathering on the mechanical, thermal, contact angle, and water absorption behavior of neat epoxy, individual kenaf and sisal, and kenaf/sisal hybrid epoxy composites. The composite was fabricated by hand layup method. Among the various composites studied, sisal/kenaf/sisal hybrid epoxy composites showed the best properties and retained the thermo-mechanical properties with the lowest water absorption properties even after the weathering test. Thus, composites with hybridized kenaf and sisal with sisal outer layer are encouraging semistructural materials in outdoor applications.

## 1. Introduction

Epoxy resins are one of the important thermosetting polymers used for making numerous materials for many applications. Recently, several studies on epoxy thermoset are focused on developing advanced composites, shape memory polymers, flexible polymer, self-healing polymers, etc. [1,2,3,4,5,6,7]. They are used for making advanced composites, adhesives, coatings, and electronic applications, due to their versatility in properties [8]. The use of synthetic fiber in epoxy resin provides a good increase in thermo-mechanical properties and it is widely used in composite industry. However, the management of these composites after use is a serious threat. Therefore, many scientists are recently focused on replacing non-recyclable synthetic fibers with more environmentally friendly materials [9]. Among the various natural fibers such as plant fibers, mineral fibers, and animal fibers, the plant fibers are a good choice for the replacement of synthetic fibers [10,11]. In fact, many industries such as automotive, civil construction, and armor systems use plant fibers for making lightweight composites [12,13,14,15].

Recently, several approaches have been adopted for the replacement of synthetic fibers, in one such approach, Giridharan [16] hybridized ramie with E-glass fibers. The composites with 20% and 30% fiber content were prepared using hand layup method. The hybrid epoxy composites show better tensile, flexural, and impact strength compared to individual composites. Among the samples prepared, 30% composites with 10% ramie and 20% E-glass fibers give the best results. Neves et al. [17] fabricated epoxy composites with hemp fiber. The composites with up to 30 vol % of continuous and aligned hemp fibers are used. The mechanical (tensile and flexural) properties of the composites showed an increase with fiber loading and the best mechanical properties are observed at 30 vol % of fiber loading. Mohan and Kanny [18] prepared nanoclay-infused banana fiber-reinforced epoxy composite cylinders. The banana fiber length used is 40 mm, while the fiber loading is fixed at 40 vol %. The composites show good compression, tensile, flexural, and short beam strength. This result showed good reinforcement of the nanoclay-infused banana fiber with epoxy matrix. Chee et al. [19] studied the viscoelastic and thermal expansion behavior of epoxy-, individual bamboo fiber non-woven mat-, individual kenaf fiber woven mat-, and hybrid bamboo/kenaf mat-reinforced hybrid composites. The among the hybrid composites, the composite with 50:50 composition showed reasonably good storage modulus. The Cole–Cole plot of this composition reveals good interfacial adhesion between the fiber and epoxy matrix. Khan et al. [20] studied the effect of non-woven kenaf mat, clay/kenaf/epoxy, modified clay/kenaf/epoxy, and cellulose nanofibers/kenaf/epoxy. Among these composites, prepared cellulose nanofibers/kenaf/epoxy gives the best mechanical properties Thus, the introduction of cellulose nanofibers in non-woven kenaf mat/epoxy composite is an innovative method to improve the performance of the composites. Mostafa et al. [21] studied the fatigue and tensile properties of glass/epoxy, 30:70 jute/glass, 45:55 jute/glass, and jute/epoxy composites. The glass/epoxy composites show the best properties and jute/epoxy composites show the least properties, while the hybrid composites show intermediate behavior. The study recommends the use of cheap eco-friendly jute as a partial replacement of glass fibers for the fabrication of epoxy composites. Saba et al. [22] fabricated epoxy hybrid composites with magnesium hydroxide (MH) and kenaf mats. The hybrid composites with 20% MH show the best tensile strength, flexural strength, impact strength, and storage modulus. Thus, the study reveals a remarkable change in the mechanical properties in the presence of MH in kenaf mat reinforced epoxy composite.

Thus, from the literature reports, natural fiber-reinforced epoxy composites have attracted increasing interest over the last few years. This is because of the many advantages of using natural fiber in epoxy composites. First, the amount of non-biodegradable epoxy resin used for the manufacturing of the products can be reduced with the incorporation of natural fiber fabric. Second, for long-term applications, the natural fiber fabric-based composites can provide better mechanical properties. Third, the reuse, recycling, or degradability of synthetic fiber after the service life is difficult since the epoxy resin is warped over it. On the other hand, natural fibers are cheap, easy to process, eco-friendly, and biodegradable. Fourth, this method facilitates the development of cheap and more eco-friendly composites. However, comparing with synthetic fibers-based composites, natural fiber-based composites are prone to degradation when used for outdoor applications due to moisture absorption, microbial attack, temperature change, and ultraviolet radiation. The water absorption of the natural fiber-reinforced composites is reported to be higher than synthetic fiber-reinforced composites; moreover, the fiber degradation in the matrix may initiate microcracks, which will eventually deteriorate the performance of the composites. To address these issues few of the recent studies are focused on the accelerated weathering analysis of natural fiber-reinforced epoxy composites [23,24,25,26]. Chee et al. [25] studied the effect of accelerated aging on the thermal properties of neat epoxy, individual bamboo mat-reinforced epoxy, kenaf mat-reinforced epoxy, and bamboo/kenaf hybrid epoxy composites. The composites showed good thermal stability and modulus even after the weathering test. In this study neat epoxy, individual kenaf, individual sisal, and hybrid kenaf/sisal fiber fabric epoxy composites with different stacking sequences have been prepared. The influence of accelerated aging on the mechanical, thermal, contact angle, and water absorption behavior of the individual and hybrid composite were studied.

## 2. Materials and Methods

**Figure 1:**
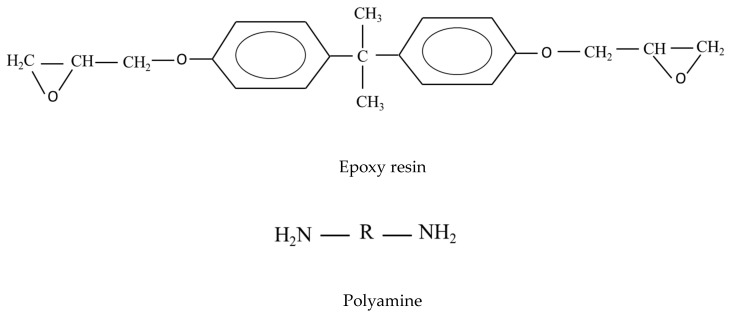
Chemical structure of epoxy resin (Epotec YD-535 LV) and hardener (Epotec TH-7257).

The epoxy resin (Epotec YD-535 LV) and hardener (Epotec TH-7257) were kindly supplied by Aditya Birla Chemicals Ltd., Rayong, Thailand. The epoxy resin has a density of 1.1–1.2 gm/cc and a viscosity of 1000–1500 mPa s at 25 °C. The Epotec TH-7257 is a polyamine-based hardener [27]. The chemical structure of epoxy resin (Epotec YD-535 LV) and hardener (Epotec TH-7257) are given in Figure 1. The kenaf and sisal plain woven fabrics were procured from Sri Lakshmi Group Exports & Imports, Guntur, Andhra Pradesh, India. The individual properties of the fibers were given in our previous publication [26]

### 2.1. Composite Manufacturing

**Figure 2:**
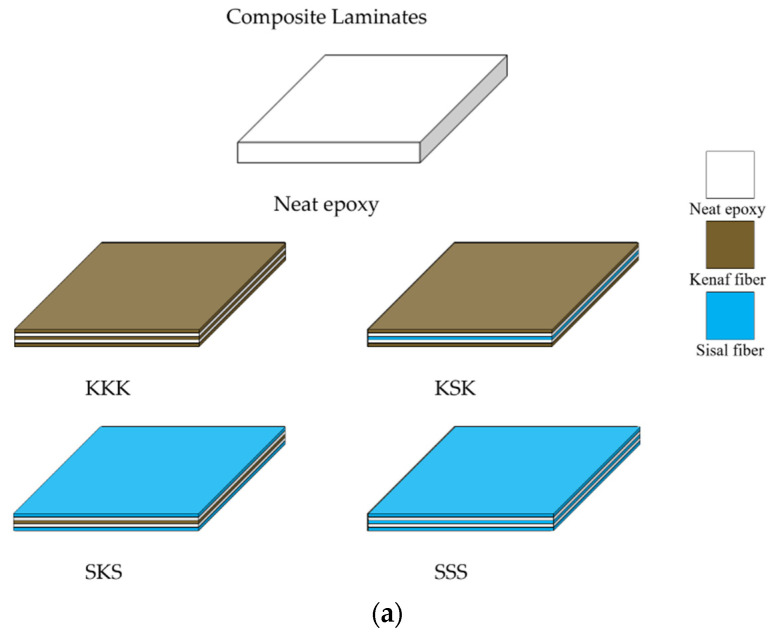

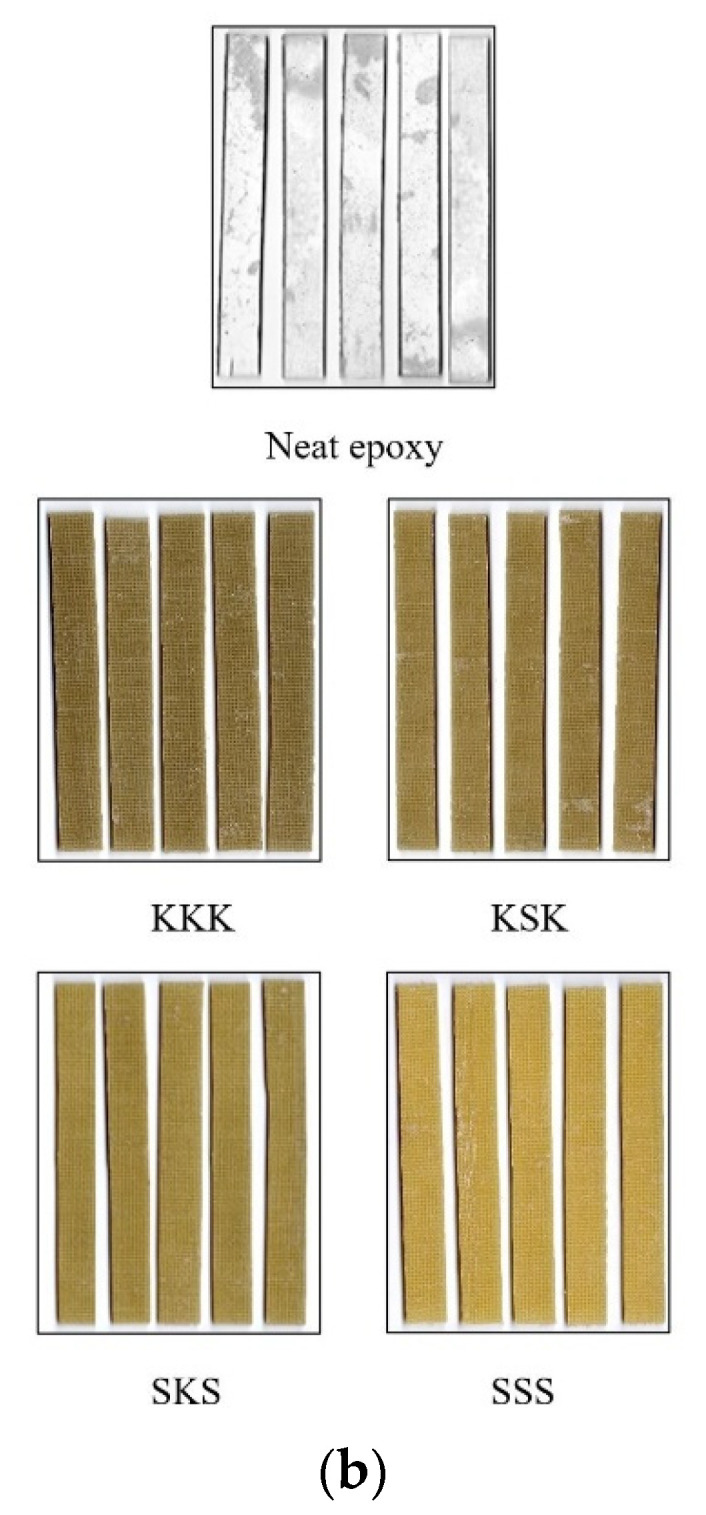
(**a**) Schematic of the composites prepared, (**b**) Photographs of composites after cutting.

The neat epoxy and epoxy composites were prepared as follows [26]. For the preparation of the neat epoxy system, the epoxy resin was mixed with the hardener in a 100:35 ratio at room temperature and poured into the mold with a dimension of 300 × 300 mm^2^ and a thickness of 3 mm. The samples were cured at room temperature for 24 h, followed by post-curing at 80 °C for 24 h. For the preparation of epoxy composites, first, the fiber fabrics were dried at 60 °C for 24 h in an air oven and then cut it to fit in the mold size of 300 × 300 mm^2^. Second, the epoxy/hardener mixture was prepared. Third, the three layers of the fabric were placed in the mold and were applied with the epoxy hardener mixture by hand layup method. This was followed by curing at room temperature for 24 h, followed by post-curing at 80 °C for 24 h. Irrespective of the composites, the fiber volume fraction used was ~18%. The average standard deviation in thickness of the prepared composites varies between 0.02 to 0.15 mm. The cured samples were later cut into small size for testing using a diamond-tipped saw cutter based on ASTM standards. Before testing, sandpaper was used to remove the defects and to make the samples more uniform. The schematic of the prepared samples and the photographs of the cut samples (before the application of sandpaper) is given in Figure 2a,b.

### 2.2. Accelerated Weathering

Epoxy composites have been used in many outdoor applications such as automobile, aerospace, and construction and building. These composites are exposed to harsh environmental conditions like temperature, humidity, and UV radiation during their service. The accelerated weathering instrument can replicate various real environmental conditions using a special weathering chamber. The instrument accelerates the weathering process and provides information on the durability of the composites. The epoxy composites are indeed coated with UV resistant paints before outdoor applications. However, in this study, the accelerated weathering test was used to predict the usefulness of epoxy fiber–fabric composites for long-term applications. As temperature, humidity, and light conditions may influence the structural properties of the composites, the accelerated weathering experiment was carried out in a Q-Sun Xe-3 weathering tester machine, Q-Lab Corporation, Westlake, Ohio, USA, according to ASTM standard G155-13 cycle 1, as in our previous study [26]. The xenon arc UV light source was used to replicate the spectrum of sunlight. In this method, the test specimens were placed inside the accelerated weathering chamber, and the samples were exposed to repeated cycles of UV light, temperature, and humidity for 555.55 h. After weathering, the samples were taken out from the chamber and were used for thermo-mechanical testing.

### 2.3. Characterization

A M1 type Comtech tensile testing machine was used for testing the mechanical properties of the samples based on ASTM D3039. The test was carried out at a loading rate of 2.5 mm/min and the load cell used was 2.5 KN. Five samples were tested for each composition and the average was taken as the final value. A Zwick/Roell pendulum impact tester was used to study the impact properties of the composite based on ASTM D256-06. Five samples were tested for each composition and the average was taken as the final value. A Hitachi TM4000Plus scanning electronic microscope was used to examine the fracture surface of the composites. The thermal stability of the composites was measured using a TGA 2-Mettler Toledo TGA/DSC 3+ HT/1600, Schwerzenbach, Switzerland. The test was carried out from 30 to 700 °C, at a heating rate of 10 °C/min. The glass transition temperature (*T*_g_) of the composites was evaluated using a DSC 3-Mettler Toledo, Schwerzenbach, Switzerland. Approximately 5 mg of the sample was tested in the temperature range −50 to 200 °C at a heating rate of 20 °C/min. The viscoelastic properties of the composites were measured using a DMA/SDTA861e, Mettler Toledo, the test was carried out from −30 to 140 °C at a heating rate of 2 K/min and frequency 1 Hz. A OCA 15LJ data physics-contact angle instrument was used to measure the contact angle of the composites. To measure the contact angle, the water droplets were placed at different positions of the same sample and an average of 6–10 measurements were taken as the contact angle value. The water absorption behavior of the composites was studied based on ASTM D570. The samples were carefully cut into a small disc of 50 mm diameter and thickness 3 mm before the water absorption studies. The edges are not isolated in this experiment. The composite samples were immersed in distilled water for 120 days and the weight change is measured for every 10 days.

## 3. Results and Discussion

### 3.1. Mechanical Properties

**Figure 3:**
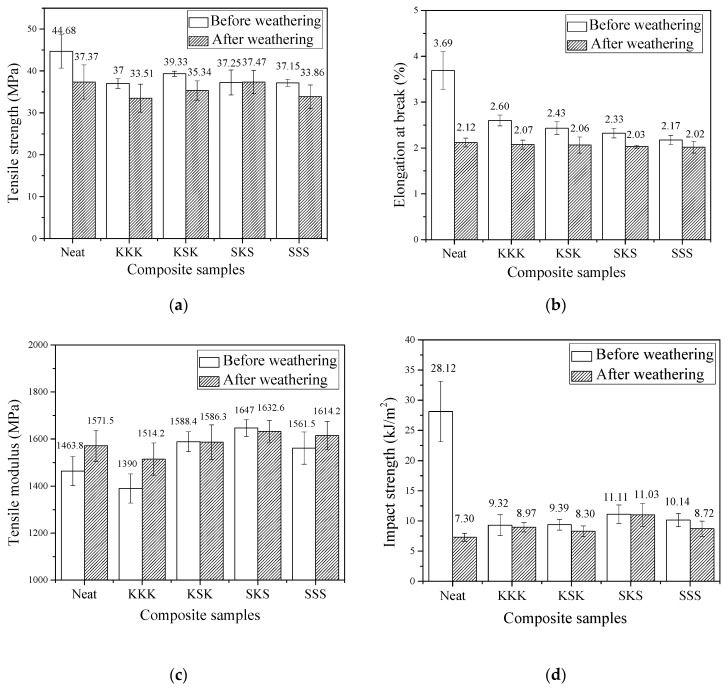
The (**a**) tensile strength, (**b**) elongation at break, (**c**) tensile modulus, and (**d**) impact strength of epoxy composite before and after weathering.

The tensile and impact properties of neat epoxy, individual kenaf fabric, individual sisal fabric, and hybrid kenaf/sisal fabric-reinforced epoxy composites before and after the weathering test have been studied. The tensile and impact results of individual and hybrid composites before and after the accelerated weathering test are given in Table 1. The comparison of the tensile properties of the epoxy composite before and after the accelerated weathering is shown in Figure 3. The tensile strength of the neat epoxy system showed a tensile strength of ~45 MPa. This value decreases to between 33 and 39 MPa with the incorporation of individual fibers and for hybrid composites. The values of tensile strength are connected with the interfacial adhesion between the fabric and polymer. The drop in tensile strength suggests an unfavorable interaction between the fabric and matrix [28]. Other factors, such as moisture absorption of the fabric and the void formation during the fabrication of composites, may have a detrimental effect on the mechanical properties of the composites [29,30]. Interestingly, the type of fabric, hybridization, and/or stacking sequence has minimum effect on the tensile strength of the epoxy system. The neat epoxy system showed a significant decrease in tensile properties after the weathering test. This is due to the polymer chain scission caused by the UV irradiation, high moisture content, and elevated temperature in the accelerating chamber during the weathering test [31]. On the other hand, the drop in tensile strength is less for the individual composites and KSK hybrid composites. The drop in tensile strength of the composites is due to the matrix degradation and fiber debonding caused by the accelerating weathering [32]. Note that the tensile strength of the SKS hybrid composites is retained even after the accelerating weathering test. Furthermore, the SKS hybrid composite shows the highest tensile strength after the accelerating weathering test.

The elongation at break of the neat epoxy system is reduced with the incorporation of individual fabrics or hybrid fabrics. The drop in elongation at break is primarily caused by the voids in the fabricated composites that may act as stress concentrators resulting in the breakage of the composites at lower applied load and strain [33]. The elongation at break of the epoxy system is reduced after the weathering test. However, only a marginal drop in elongation at break is observed for individual or hybrid composites. After the weathering test, the neat epoxy system and composites show approximately the same elongation at break. The tensile modulus of the neat epoxy system is 1464 MPa. The composites show fluctuations in tensile modulus with the lowest for KKK and the highest for SKS. On the other hand, the modulus of the neat epoxy system and the individual composites are increased after weathering studies. This may be due to the embrittlement of the epoxy matrix caused by the accelerated weathering [26]. However, the hybrid composites show only marginal changes in the modulus before and after the weathering test. The impact strength of the neat epoxy system is 28.12 MPa, and the incorporation of fabrics into the epoxy matrix reduced the impact strength. The drop in impact strength suggests unfavorable interfacial interactions between the fabrics and epoxy matrix [34]. On the other hand, for the composites, the impact strength is retained even after the weathering test and the obtained impact strength values are more than the neat epoxy system. Moreover, it is worth mentioning that the SKS hybrid composite shows the highest impact strength after the weathering test. Thus, we were relieved that after the weathering test, the SKS hybrid composites showed better mechanical properties compared to the neat epoxy system and other composites fabricated. It can be suggested that the hybrid composites with sisal outer layer and kenaf inner layer may be used long term semi-structural applications since the mechanical properties are not affected by aging studies.

**Figure 4:**
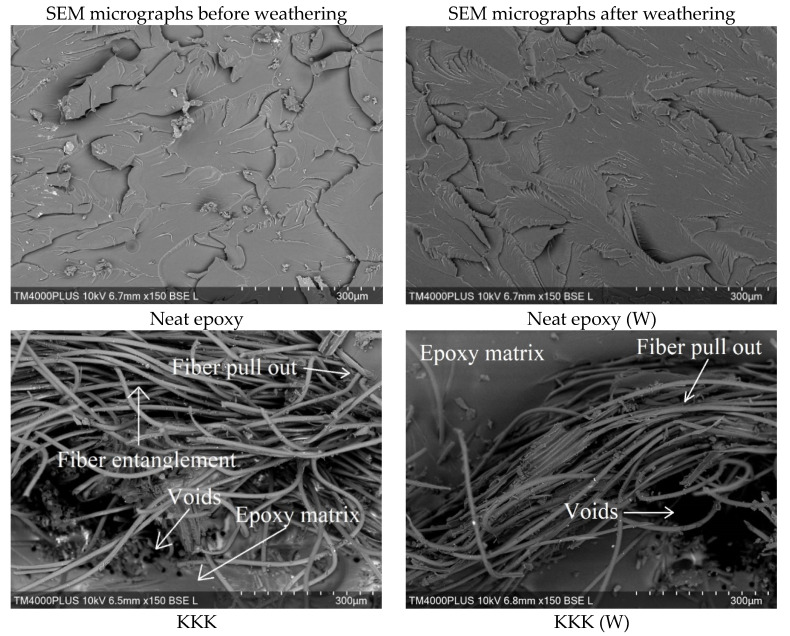

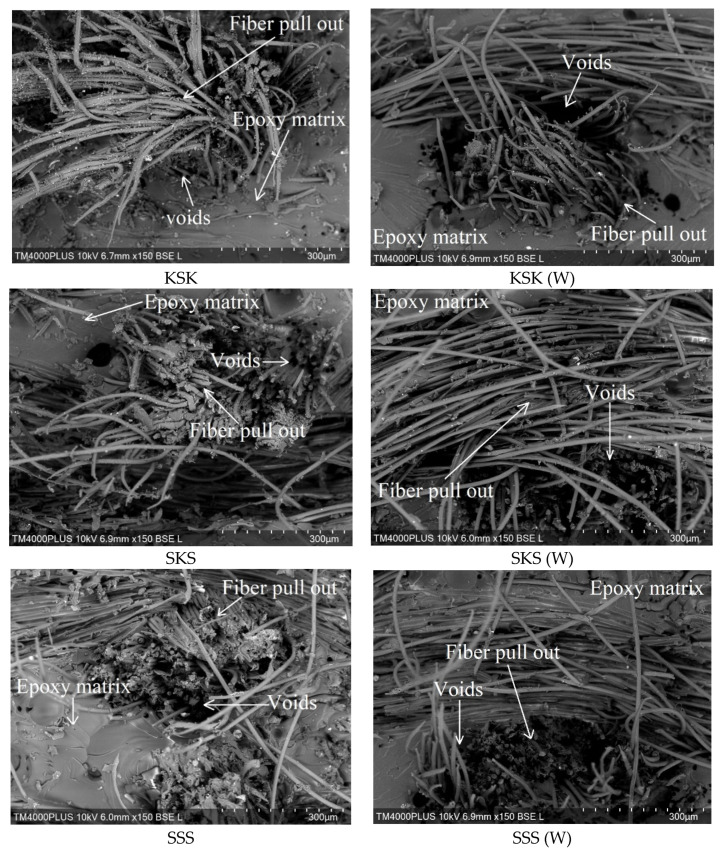
SEM micrographs of the tensile fracture samples before and after weathering.

The SEM micrographs of the tensile fracture surface of the neat epoxy system and its composites before and after aging studies are shown in Figure 4. The neat epoxy system irrespective of the aging studies showed river like brittle fractures throughout the epoxy matrix. SEM micrographs of the composites before aging studies show lack of epoxy resin adhesion to fibers, with multiple fiber pull out, fiber entanglement, and voids. The poor interfacial adhesion between the fiber and the epoxy matrix is because of the unfavorable interactions caused by the moisture absorption of the fabric and the void formation during the fabrication of composites. Thus, it can be substantiated that the drop in mechanical properties of the fiber-reinforced composites is due to the poor interfacial adhesion between the fiber fabric and the epoxy matrix [32]. On the other hand, the SEM micrographs of the composites after aging studies show more fiber pullout and larger void formation irrespective of the stacking sequence. This suggests deterioration in interfacial adhesion between the polymer and fabric due to the matrix degradation. Thus, the drop in the mechanical properties of the composites after the accelerated weathering is due to the epoxy matrix degradation caused by the accelerated weathering.

### 3.2. Thermal Studies

**Figure 5:**
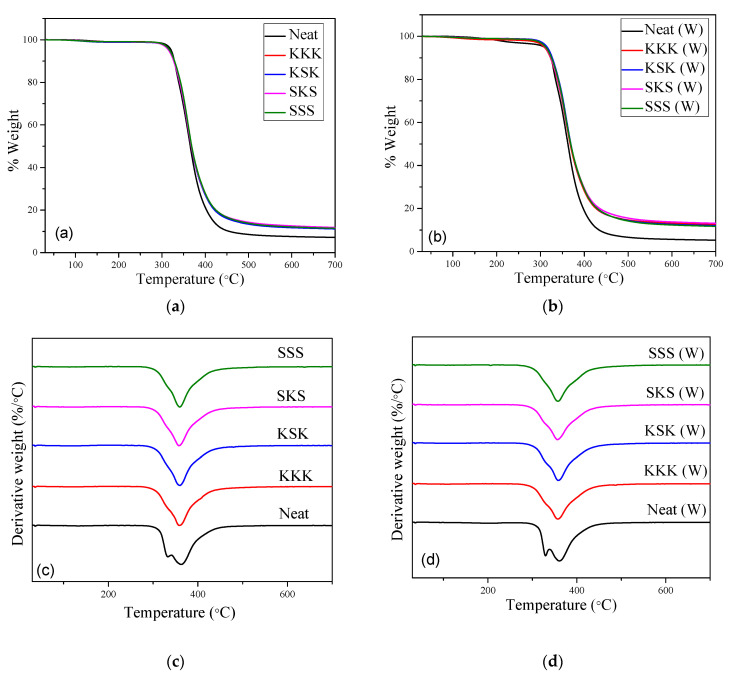
Thermogravimetric analysis (TGA) curves of (**a**) before and (**b**) after weathering composite samples. Differential thermogravimetry (DTG) curves of (**c**) before and (**d**) after weathering composite samples.

The thermogravimetric analysis of the neat epoxy, individual composites, and hybrid composites before and after weathering studies is shown in Figure 5a,b. The onset decomposition temperature (*T*_on_), final decomposition temperature (*T*_f_), and maximum decomposition temperature (*T*_max_) obtained from the thermogram are given in Table 2. The main degradation of the neat epoxy, individual composites, and hybrid composites is due to the epoxy chain scission at high temperatures [35]. The *T*_on_, *T*_max_, and *T*_f_ values of composites before weathering are stable with only marginal changes with the incorporation of fiber fabrics. Similarly, irrespective of the introduction of the fibers, fiber type, hybridization, and stacking sequence, the neat epoxy and epoxy composites show no major change in the values of *T*_on_, *T*_max_, and *T*_f_ after the weathering test. These results prove that the composites are thermally stable before and after the weathering test. It is important to point out that the neat epoxy system and composites show two peaks: a shoulder and the main peak in the DTG curve (Figure 5c,d). The shoulder is due to the decomposition of lower molecular weight sites and while the main peak is the decomposition of highly crosslink density sites of epoxy matrix [36].

**Figure 6:**
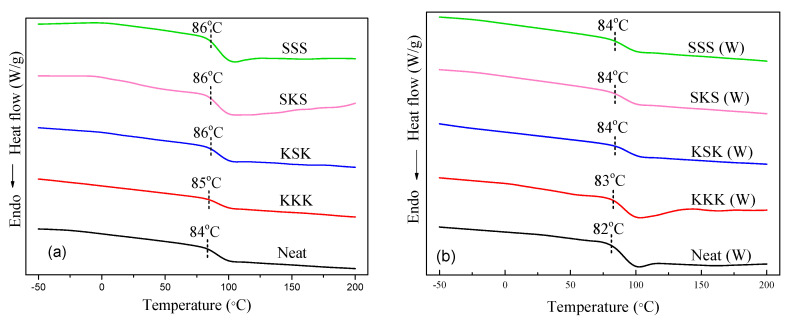
DSC thermograms of (**a**) before and (**b**) after weathering composite samples.

The *T*_g_ of neat epoxy, individual composites, and hybrid composites before and after weathering studies has been studied. The DSC thermogram of the composites is shown in Figure 6. The *T*_g_ of neat epoxy is observed at 84 °C, and this value marginally increased to higher temperatures for the individual and hybrid composites. The increase in *T*_g_ may be due to the restriction in the mobility of the polymer chains caused by the fiber fabrics [37]. On the other hand, the *T*_g_ is marginally reduced after the aging studies, which may be due to the polymer degradation caused during the weathering studies. 

### 3.3. Contact Angle Measurement

**Figure 7:**
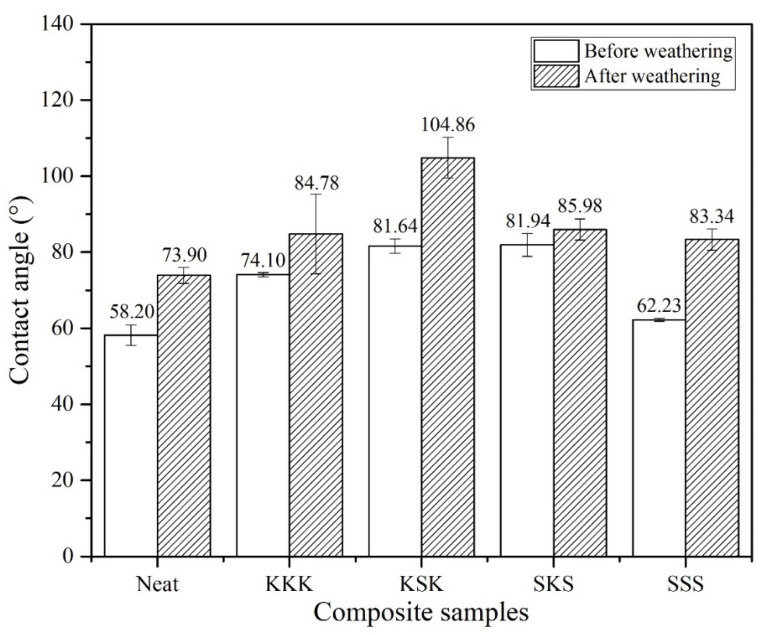
Comparison of contact angle measurements of before and after weathering composite samples.

The contact angle values are a measure of the hydrophilicity/hydrophobicity of the composites. It gives information on the surface properties and wettability of the polymer surface. The superhydrophobic surface (contact angle greater than 150°) repels water and reduces the water absorption. Similarly, if the contact angle is less than 10°, the composite will be superhydrophilic with higher wettability and good self-cleaning ability [38,39,40]. For outdoor applications, the composite structures are usually coated with paint, and for coating hydrophilic surfaces are preferred. The comparison of the contact angle measurements of neat epoxy, individual composites, and hybrid composites before and after weathering composite samples is shown in Figure 7. The neat epoxy system shows a contact angle of 58°, typical for a hydrophilic material, and water can easily spread over it. However, the composites show higher contact angle values, and the maximum contact angle was observed for SKS composites but still in the hydrophilic range. This shows that the interaction between the water and epoxy surface became less in composites compared to the neat epoxy polymer. The contact angle depends on the surface roughness of the composites. The surface of the neat epoxy system is smooth, while the surface of the composites is rough due to the presence of fabrics at the composite surface and that is probably the reason for the increase in contact angle values of the composites. Interestingly, the contact angle value further increases for all the samples after the weathering test. These results are in agreement with our previous study [26]. The increase in contact angle is due to the surface roughness of the composites due to the surface degradation caused during the accelerated weathering. The maximum contact angle is shown by weathered KSK hybrid composite (104.86°) and shows a hydrophobic behavior. That means the water droplets may roll over KSK surface rather than spreading or wetting.

### 3.4. Water Absorption Behavior

**Figure 8:**
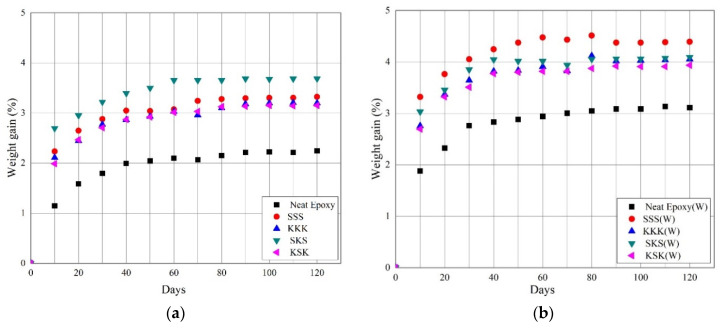
Water absorption behavior of the composites (**a**) before and (**b**) after weathering.

The water absorption behavior of the neat epoxy, individual composites, and hybrid composites before and after weathering composite samples was studied up to 120 days. The water absorption behavior of the composites before and after weathering is shown in Figure 8. In the initial days of the experiment, the water absorption increases rapidly, followed by a slowdown and then a plateau region [41]. The weight gain is minimum for the neat epoxy system and is ~2%. However, for composites, it is more than 3%. This is due to the presence of fiber fabrics in the epoxy matrix. The voids generated at the fabric/matrix interface, crack formation in the matrix, hollow structure of fibers, and the hydroxyl functional groups present in the fibers influence the water uptake of the composites [26,41,42]. After weathering, the water absorption of the neat epoxy system increases from ~2% to ~3%, which may be due to the formation of the microcracks in the matrix, and thus water enters through the microcracks and water absorption increases. Similarly, the composites also show higher water absorption from ~3% to 4% and above. The formation of microcracks and the degradation of fibers created new path for water transport and hence higher water absorption. Thus, one of the limitations of using fiber fabric-based epoxy composite is its water absorption properties.

### 3.5. Dynamic Mechanical Analysis (DMA)

**Figure 9:**
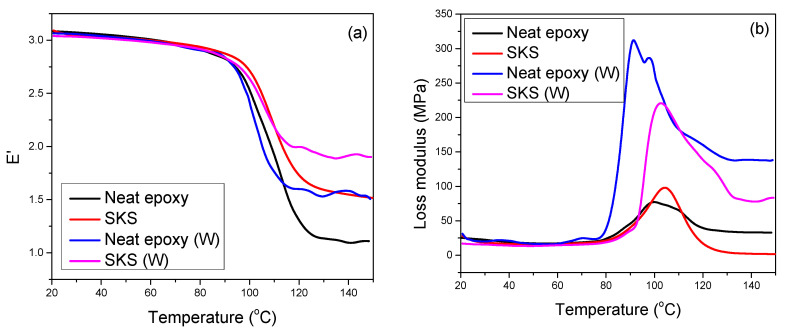
(**a**) Storage modulus and (**b**) loss modulus curves of neat epoxy and SKS composite before and after weathering.

Among the various composites studied, the SKS hybrid composites show reasonably good thermal stability, mechanical properties, and water absorption properties. Therefore, the viscoelastic properties of neat epoxy and SKS hybrid composites before and after weathering samples are studied. The viscoelastic properties of neat epoxy and SKS composite before and after weathering are shown in Figure 9. The SKS hybrid composites show storage modulus consistent with neat epoxy. That means the incorporation of fiber fabrics in the epoxy matrix has no effect on the storage modulus. Interestingly, the accelerated aging of the composite samples has minimum effect on the storage modulus in the glassy state. The sharp drop in storage modulus at 100 °C is due to the *T*_g_. The *T*_g_ is followed by the rubbery region [43]. As in our previous publication [26], the drop in storage modulus at the *T*_g_ is maximum for neat epoxy resin, but the drop is not very pronounced for the composites before and after aging. This is due to the presence of stiffer fiber fabric in the rubbery region. 

The reinforcement effect of fiber fabric with the epoxy matrix can be measured by calculating the reinforcement effectiveness concept (C) the using the Equation (1) [44] and the obtained values are given in Table 3.

(1) C=(E′G/E′R)Composite(E′G/E′R)Resin

*E*′*_G_* = storage modulus at the glassy state (at 35 °C)
*E*′*_R_* = storage modulus at the rubbery state (at 120 °C).

If the value of C is less than 1, it represents the interaction between fabric and epoxy matrix. The C value of SKS composite is less than 1, which shows the existence of the interaction between fabric and epoxy matrix. As there is no increase in the modulus of the epoxy glassy state with the incorporation of the fabrics, rather than the reinforcement effect, the stiffer fiber fabric in the rubbery region could be the cause for the lower C value.

The loss modulus profile of the neat epoxy system and SKS composite is shown in Figure 9b. The SKS composites (before weathering) show a marginal improvement in *T*_g_ with the incorporation of fiber fabric. The neat epoxy system after the weathering test shows lower *T*_g_ and higher loss modulus peak height. The high loss modulus peak values showed that the intermolecular bonding is destroyed during the accelerated weathering test [45]. It is worth to mention that for SKS composite the *T*_g_ was not affected with the accelerated weathering test; moreover, the loss modulus peak height is lower when compared with neat epoxy system showing that the fiber fabrics prevent the degradation of the epoxy matrix at the macro-level.

## 4. Conclusions

This study aimed at investigating the influence of accelerated weathering on the performance of natural fiber fabric-reinforced epoxy composites. The mechanical, thermal, morphology, contact angle, and water absorption properties were evaluated. Among the composites prepared, hybrid SKS composites showed reasonably good thermo-mechanical, contact angle, and water absorption properties. From the obtained results, it can be suggested that the hybridization of sisal and kenaf fiber fabrics may be used for the fabrication of semi-structural epoxy composites.

## Figures and Tables

**Table 1:** The tensile and impact properties of epoxy composite before and after weathering.

Composites	Tensile Strength (MPa)	Elongation at Break (%)	Tensile Modulus (MPa)	Impact Strength KJ/m^2^
Neat	44.68 ± 4.00	3.69 ± 0.41	1463.8 ± 61.5	28.12 ± 5.02
KKK	37.00 ± 1.16	2.60 ± 0.12	1390.0 ± 61.9	9.32 ± 1.72
KSK	39.33 ± 0.62	2.43 ± 0.14	1588.4 ± 42.3	9.39 ± 0.9
SKS	37.25 ± 2.98	2.33 ± 0.11	1647.0 ± 36.3	11.11 ± 1.53
SSS	37.15 ± 0.86	2.17 ± 0.10	1561.5 ± 68.2	10.14 ± 1.11
Neat (W)	37.37 ± 4.08	2.12 ± 0.10	1571.5 ± 66.0	7.30 ± 0.67
KKK (W)	33.51 ± 3.33	2.07 ± 0.10	1514.2 ± 68.9	8.97 ± 0.79
KSK (W)	35.34 ± 2.30	2.06 ± 0.18	1586.3 ± 73.4	8.30 ± 0.88
SKS (W)	37.47 ± 2.80	2.03 ± 0.03	1632.6 ± 46.1	11.03 ± 1.91
SSS (W)	33.86 ± 2.79	2.02 ± 0.13	1614.2 ± 60.3	8.72 ± 1.25

**Table 2:** *T*_on_, *T*_max_, and *T*_f_ of before and after weathering composite samples.

Composites	(*T*_on_)	(*T*_max_)	(*T*_f_)	Char Residue (700 °C)
Neat	326.74	362.33	395.47	7.17
KKK	329.03	359.50	392.89	11.27
KSK	329.76	361.00	391.30	11.12
SKS	328.97	356.33	391.45	11.83
SSS	331.42	359.83	393.92	11.43
Neat (W)	326.02	360.50	395.53	5.24
KKK (W)	325.91	356.50	395.59	12.57
KSK (W)	328.50	358.17	397.13	12.03
SKS (W)	325.53	356.17	396.14	13.12
SSS (W)	327.10	358.00	395.17	11.67

**Table 3:** Reinforcement effectiveness (C) of neat epoxy and SKS composite.

Samples	C
Neat epoxy	1
SKS	0.35
Neat epoxy (W)	1
SKS (W)	0.38

## References

[1] Liu Y., Guo Y., Zhao J., Chen X., Zhang H., Hu G., Yu X., Zhang Z. (2019). Carbon fiber reinforced shape memory epoxy composites with superior mechanical performances. Compos. Sci. Technol..

[2] Guo Y., Liu Y., Liu J., Zhao J., Zhang H., Zhang Z. (2020). Shape memory epoxy composites with high mechanical performance manufactured by multi-material direct ink writing. Compos. Part A Appl. Sci. Manuf..

[3] Altuna F., Hoppe C., Williams R. (2016). Shape memory epoxy vitrimers based on DGEBA crosslinked with dicarboxylic acids and their blends with citric acid. RSC Adv..

[4] Meng Q., Zhao Y., Liu Z., Han S., Lu S., Liu T.M. (2019). Flexible strain sensors based on epoxy/graphene composite film with long molecular weight curing agents. J. Appl. Polym. Sci..

[5] Huang J., Nie X. (2016). A simple and novel method to design flexible and transparent epoxy resin with tunable mechanical properties. Polym. Int..

[6] Guadagno L., Vertuccio L., Naddeo C., Calabrese E., Barra G., Raimondo M., Sorrentino A., Binder W.H., Michael P., Rana S. (2019). Self-healing epoxy nanocomposites via reversible hydrogen bonding. Compos. Part B Eng..

[7] Yuan D., Bonab V.S., Patel A., Manas-Zloczower I. (2018). Self-healing epoxy coatings with enhanced properties and facile processability. Polymer.

[8] Parameswaranpillai J., Hameed N., Pionteck J., Woo E.M. (2017). Handbook of Epoxy Blends.

[9] Rajak D.K., Pagar D.D., Menezes P.L., Linul E. (2019). Fiber-reinforced polymer composites: Manufacturing, properties and applications. Polymers.

[10] Hassan T., Jamshaid H., Mishra R., Khan M.Q., Petru M., Novak J., Choteborsky R., Hromasova M. (2020). Acoustic, mechanical and thermal properties of green composites reinforced with natural fibers waste. Polymers.

[11] Wang H., Hassan E., Memon H., Elagib T., Abad AllaIdris F. (2019). Characterization of natural composites fabricated from Abutilon-fiber-reinforced Poly (Lactic Acid). Processes.

[12] Prasad S.V.N.B., Kumar G.A., Sai K.V.P., Nagarjuna B. Design and optimization of natural fibre reinforced epoxy composites for automobile application. Proceedings of the AIP Conference, Bannari Amman Institute of Technology.

[13] Benzarti K., Chlela R., Quiertant M., Zombre W., Curtil L. Durability of flax/bio-epoxy composites intended for structural strengthening. Proceedings of the MATEC Web of Conference.

[14] Kumar G.R., Hariharan V., Saravanakumar S.S. (2019). Enhancing the free vibration characteristics of epoxy polymers using sustainable phoenix sp. fibers and nano-clay for machine tool applications. J. Nat. Fibers.

[15] Garcia Filho F.D.C., Oliveira M.S., Pereira A.C., Nascimento L.F.C., Matheus J.R.G., Monteiro S.N. (2020). Ballistic behavior of epoxy matrix composites reinforced with piassava fiber against high energy ammunition. J. Mater. Res. Technol..

[16] Giridharan R. (2019). Preparation and property evaluation of Glass/Ramie fibers reinforced epoxy hybrid composites. Compos. Part B Eng..

[17] Neves A.C.C., Rohen L.A., Mantovani D.P., Carvalho J.P.R.G., Vieira C.M.F., Lopes F.P.D., Simonassi N.T., da Luz F.S., Monteiro S.N. (2020). Comparative mechanical properties between biocomposites of Epoxy and polyester matrices reinforced by hemp fiber. J. Mater. Res. Technol..

[18] Mohan T.P., Kanny K. (2019). Compressive characteristics of unmodified and nanoclay treated banana fiber reinforced epoxy composite cylinders. Compos. Part B Eng..

[19] Chee S.S., Jawaid M., Sultan M.T.H., Alothman O.Y., Abdullah L.C. (2019). Thermomechanical and dynamic mechanical properties of bamboo/woven kenaf mat reinforced epoxy hybrid composites. Compos. Part B Eng..

[20] Khan A., Asiri A.M., Jawaid M., Saba N., Inamuddin (2020). Effect of cellulose nano fibers and nano clays on the mechanical, morphological, thermal and dynamic mechanical performance of kenaf/epoxy composites. Carbohydr. Polym..

[21] Mostafa N.H. (2019). Tensile and fatigue properties of Jute-Glass hybrid fibre reinforced epoxy composites. Mater. Res. Express.

[22] Saba N., Alothman O.Y., Almutairi Z., Jawaid M. (2019). Magnesium hydroxide reinforced kenaf fibers/epoxy hybrid composites: Mechanical and thermomechanical properties. Constr. Build. Mater..

[23] Kashyap S., Nath D., Das D. (2020). Characterization, weathering and modeling of natural fibre based composites. Mater. Today Proc..

[24] Pulikkalparambil H., Rangappa S.M., Krishnasamy S., Radoor S., Hameed N., Siengchin S., Parameswaranpillai J. (2020). Accelerated weathering studies of bioepoxy/ionic liquid blends: Influence on physical, thermo-mechanical, morphology and surface properties. Mater. Res. Express.

[25] Chee S.S., Jawaid M., Sultan M.T.H., Alothman O.Y., Abdullah L.C. (2019). Accelerated weathering and soil burial effects on colour, biodegradability and thermal properties of bamboo/kenaf/epoxy hybrid composites. Polym. Test..

[26] Yorseng K., Rangappa S.M., Pulikkalparambil H., Siengchin S., Parameswaranpillai J. (2020). Accelerated weathering studies of kenaf/sisal fiber fabric reinforced fully biobased hybrid bioepoxy composites for semi-structural applications: Morphology, thermo-mechanical, water absorption behavior and surface hydrophobicity. Constr. Build. Mater..

[27] Kanchanomai C., Thammaruechuc A. (2009). Effects of stress ratio on fatigue crack growth of thermoset epoxy resin. Polym. Degrad. Stab..

[28] Liu X.Y. (2007). Surface modification and micromechanical properties of jute fiber mat reinforced polypropylene composites. Express Polym. Lett..

[29] Parbin S., Waghmare N.K., Singh S.K., Khan S. (2019). Mechanical properties of natural fiber reinforced epoxy composites: A review. Procedia Comput. Sci..

[30] Ismail A.S., Jawaid M., Naveen J. (2019). Void content, tensile, vibration and acoustic properties of kenaf/bamboo fiber reinforced epoxy hybrid composites. Materials.

[31] Jawaid M., Saba N., Alothman O.Y., Tahir P.M. Effect of accelerated environmental aging on tensile properties of oil palm/jute hybrid composites. Proceedings of the AIP Conference.

[32] Chang L.N., Chow W.S. (2010). Accelerated weathering on glass fiber/epoxy/organo-montmorillonite nanocomposites. J. Compos. Mater..

[33] Sogancioglu M., Yel E., Ahmetli G. (2020). Behaviour of waste polypropylene pyrolysis char-based epoxy composite materials. Environ. Sci. Pollut. Res..

[34] Sanjay M.R., Arpitha G.R., Senthamaraikannan P., Kathiresan M., Balaji S., Yogesha B. (2018). The hybrid effect of Jute/Kenaf/E-glass woven fabric epoxy composites for medium load applications: Impact, inter-laminar strength, and failure surface characterization. J. Nat. Fibers.

[35] Liu M., Guo B., Du M., Lei Y., Jia D. (2008). Natural inorganic nanotubes reinforced epoxy resin nanocomposites. J. Polym. Res..

[36] Nuruddin M., Hosur M., Mahdi T., Jeelani S. (2017). Flexural, viscoelastic and thermal properties of epoxy polymer composites modified with cellulose nanofibers extracted from wheat straw. Sens. Transducers.

[37] Khan Z.I., Arsad A., Mohamad Z., Habib U., Zaini M.A.A. (2020). Comparative study on the enhancement of thermo-mechanical properties of carbon fiber and glass fiber reinforced epoxy composites. Mater. Today Proc..

[38] Rathod V.T., Kumar J.S., Jain A. (2017). Polymer and ceramic nanocomposites for aerospace applications. Appl. Nanosci..

[39] Puliyalil H., Filipič G., Cvelbar U. (2019). Selective plasma etching of polymers and polymer matrix composites. Non-Thermal Plasma Technology for Polymeric Materials.

[40] Gladunova O.I., Fedorova Y.E., Astashkina O.V., Lisenko A.A. (2015). Composites with hydrophobic surfaces. Fibre Chem..

[41] Alamri H., Low I.M. (2012). Mechanical properties and water absorption behaviour of recycled cellulose fibre reinforced epoxy composites. Polym. Test..

[42] Muñoz E., García-Manrique J.A. (2015). Water Absorption Behaviour and Its Effect on the Mechanical Properties of Flax Fibre Reinforced Bioepoxy Composites. Int. J. Polym. Sci..

[43] Goertzen W.K., Kessler M.R. (2007). Dynamic mechanical analysis of carbon/epoxy composites for structural pipeline repair. Compos. Part B Eng..

[44] Ornaghi H.L., Neves R.M., Monticeli F.M., Almeida J.H.S. (2020). Viscoelastic characteristics of carbon fiber-reinforced epoxy filament wound laminates. Compos. Commun..

[45] Boparai K.S., Singh R. (2018). Thermoplastic composites for fused deposition modeling filament: Challenges and applications. Reference Module in Materials Science and Materials Engineering.

